# The genetic variation rs12143842 in *NOS1AP* increases idiopathic ventricular tachycardia risk in Chinese Han populations

**DOI:** 10.1038/s41598-017-08548-z

**Published:** 2017-08-21

**Authors:** Ronfeng Zhang, Feifei Chen, Honjiu Yu, Lianjun Gao, Xiaomeng Yin, Yingxue Dong, Yanzong Yang, Yunlong Xia

**Affiliations:** First Affiliated Hospital of Dalian Medical University, Dalian, China

## Abstract

Genome-wide association studies identified that the common T of rs12143842 in NOS1AP is associated with a QT/QTc interval in European populations. In this study, we test the association between the variation rs12143842 in NOS1AP and idiopathic ventricular tachycardia (IVT). A case-control association study examining rs12143842 was performed in two independent cohorts. The Northern cohort enrolled 277 IVT patients and 728 controls from a Chinese Gene ID population. The Central cohort enrolled 301 IVT patients and 803 matched controls. Genotyping was performed using high-resolution melt analysis. The minor T allele of the rs12143842 SNP was significantly associated with decreased IVT risk in the Northern cohort (adjusted *P* = 0.024, *OR* 0.71(0.52~0.96)), and this association was replicated in an independent Central Gene ID cohort (adjusted *P* = 0.029, *OR* 0.78 (0.62~0.97)). The association was more significant in the combined population (adjusted *P* = 0.001, *OR* 0.76 (0.64~0.90)). The *P* values for the genotypic association were significant for the dominant (*P* < 0.001) and additive (*P* = 0.001) models. The minor T allele for the SNP rs12143842 in *NOS1AP* is significantly associated with IVT. *NOS1AP* might be a novel gene affecting IVT, and further functional studies should be performed.

## Introduction

Idiopathic ventricular tachycardia (IVT) is defined as a set of ventricular arrhythmias that occur after ruling out structural or pathological causes^1^. Approximately 60–80% of idiopathic tachycardia originates from the right ventricular outflow tract (RVOT), and 10% originates from the idiopathic left ventricular tachycardia (ILVT). Long QT syndrome (LQTS) is characterized by abnormally prolonged QT intervals (corrected QT interval > 440 ms in men and > 460 ms in women) and is an inherited disorder in which the prolonged QT interval causes ventricular tachycardia^2^. A prolonged electrocardiographic QT interval duration, which is a measure of myocardial repolarization time, is a risk factor for idiopathic and drug-induced arrhythmias, especially ventricular arrhythmias.

Recent genome-wide association studies (GWAS) have identified several genetic loci associated with the QT interval and QTc interval. Two independent GWAS revealed two SNPs (rs12143842 and rs10494366) in the NOS1AP gene on chromosome 1q23 that are associated with QT interval in various populations of European ancestry^3, 4^. Liu J *et al*. also observed associated of the genetic variant rs12143842 in NOS1AP with the QT interval duration in a study examining a Chinese cohort^5^. Rs12143842 was also a significant predictor of prolonged repolarization^6, 7^. Variants at the NOS1AP locus confer a ~30% increased risk of sudden cardiac death (SCD) in the general population^8, 9^, and NOS1AP has also been associated with sudden cardiac arrest due to ventricular tachycardia/ventricular fibrillation in patients with coronary artery disease^10^.

Because QT prolongation increases the incidence of ventricular tachycardia, the direct association of rs12143842 with IVT should be explored further. We therefore performed a large-scale case-control association study with 578 IVT patients and 1,531 non-IVT controls in a Chinese Han Gene ID population to examine whether the NOS1AP SNP rs12143842 is associated with IVT.

## Methods

### Study subjects

The study subjects were from the Gene ID population, which is a large current Chinese database with clinical data and tissue samples from >30,000 Chinese patients and controls that can be used to identify susceptibility genes for various cardiovascular diseases. All of the subjects were of Han ethnic origin by self-description. The studies were approved by appropriate local institutional review boards for human subjects and conformed to the guidelines set forth by the Declaration of Helsinki. Written informed consent was obtained from the participants.

The case-control association study for IVT included 2 independent cohorts. The Gene ID Northern cohort included 277 IVT patients and 728 matched controls enrolled from hospitals in the northern part of China. The Gene ID Central cohort consisted of 301 cases and 803 controls enrolled from hospitals in central China. IVT diagnosis and characteristics were based on standard diagnostic criteria^1^. Any patients with structural heart disease, heart failure, coronary heart disease (CAD), stroke, long QT syndrome, and Brugada syndrome were excluded. For the control group, each subject was evaluated by ECG/Holter, and the echocardiogram did not detect any cardiac arrhythmia (e.g., atrial fibrillation, sick sinus syndrome, atrioventricular conduction block or intra-ventricular block) or structural heart diseases.

### SNP rs12143842 genotyping

Blood samples were drawn from study participants and used for genomic DNA isolation using the Wizard® Genomic DNA Purification Kit (Promega Corporation, Madison, WI, USA) according to the manufacturer’s protocol.

SNP rs12143842 was genotyped using a Rotor-Gene TM6000 High Resolution Melt system (Corbett Life Science, Concorde, NSW, Australia). Genotyping was performed in a total PCR volume of 25 μL containing 1 μL LC Green dye, 5 pmol each primer, 25 ng genomic DNA, 2.5 μL 10 × PCR buffer with 1.5 mmol/L MgCl_2_, 5 mmol deoxynucleotide triphosphate, and 1 U Taq polymerase. The thermal profile was as follows: 95 °C for 5 min; 40 cycles of 95 °C for 10 s, the annealing temperature (58.7 °C for rs12143842) for 10 s, and 72 °C for 15 s; and a final cycle of 72 °C for 10 min. Two positive controls for each genotype (T/T, T/C, and C/C) were included in each run. A total of 48 cases and controls were randomly selected to verify the genotyping results using direct DNA sequence analysis. DNA sequence analysis was performed with forward and reverse primers using the Big Dye Terminator v3.1 Cycle Sequencing Kit on an ABI PRISM 3100 Genetic Analyzer (Applied Biosystems, Foster City, CA, USA).

Primer sequences (5′-3′):

F: aactggatattaaactttgcaaaagaga; R: ttactgtgctctaaaatatgctctttatg

### Statistical analysis

SNP rs12143842 genotypes were tested for Hardy–Weinberg equilibrium among the controls using PLINK v1.05. Allelic and genotypic association of rs12143842 with IVT was assessed using Pearson’s 2 × 2 contingency table* ×* ^2^ test (PLINK v1.05). The odds ratios (ORs) and 95% confidence intervals (CIs) were estimated using the × ^2^ test (PLINK v1.05). Multivariate logistic regression analysis was performed using SPSS version 13.0 by adjusting for age and gender. *T* test analysis was performed using SPSS version 13.0. Empirical *P* values were determined using the PLINK v1.05 program with 1,000 Monte-Carlo simulations.

## Results

### Clinical characteristics

There was no deviation from the Hardy-Weinberg equilibrium for SNP rs12143842 in the control groups (*P* > 0.05; Supplementary Table 1). We performed a case control study to test the association between SNP rs12143842 and IVT in the two cohorts. The Northern cohort included 277 IVT patients and 728 controls (Table 1). The mean age of the patients was 37 ± 15 years, and 157 of them were women. The Central cohort included 301 IVT patients and 803 controls (Table 1). The mean age of the patients was 57 ± 20 years, and 114 of them were women. The IVT patients were divided into three groups according to the site of origin. In the Northern cohort, 118 patients (43.6%) originated from the left ventricle, 140 from the right ventricle, and 18 patients had unknown origin sites. In the Central cohort, 83 patients (32.0%) originated from the left ventricle, 138 patients originated from the right ventricle, and 38 patients had unknown origin sites. Table 1 shows the clinical characteristics of the study subjects.

**Table 1:** Clinical characteristics of the study population.

Characteristics	Gene ID Northern		Gene ID Central	
	IVT	Control	IVT	Control
Total number of samples	277	728	301	803
Age, years	37 ± 15	47 ± 14	57 ± 20	62 ± 12
Sex (male/female)	120/157	486/240	145/114	375/312
Category				
ILVT (%)^&^	118(42.6%)	N/A	83(32.0%)	N/A
RVOT (%)^@^	140(50.5%)	N/A	138(53.3%)	N/A
Other VT (%)	19 (6.9%)	N/A	38(14.7%)	N/A
Hypertension (%)^$^	N/A	90(12.3%)	N/A	355(51.7%)

Data are shown as the means ± SD for quantitative variables and as n (%) for qualitative variables.

*Age at first diagnosis of the disease.

^&^ILVT: idiopathic left ventricular tachycardia.

^@^RVOT: right ventricular outflow tract tachycardia.

^$^Hypertension was defined as a systolic blood pressure of >140 mmHg or a diastolic blood pressure of >90 mmHg.

### Allelic association of SNP rs12143842 with IVT

There was no deviation from the Hardy–Weinberg equilibrium for SNP rs12143842 in the control groups (P > 0.05; Supplementary Table 1). In the Chinese Gene ID Northern cohort, we genotyped 277 IVT patients with a T ratio of 27.8% and 726 controls with a T ratio of 34.7% (Supplementary Table 2). We observed a significant association between rs12143842 and IVT (*OR* = 0.73(0.58~0.90); *P-ob*s = 0.003; *P-adj* = 0.024; *P-emp* = 0.033) as shown in Table 2. Because the association between rs12143842 and IVT was novel and not observed previously, it must be replicated in an independent cohort. Thus, we performed a replication study with the Chinese Gene ID Central cohort that included 301 patients with IVT and 803 controls. The T allele of rs12143842 was associated with a significant IVT risk in the Central cohort (*OR* = 0.80(0.64~0.99); *P-ob*s = 0.045; *P-adj* = 0.029; *P-emp* = 0.022). When the Gene ID Northern and Gene ID Central cohorts are combined, the *P* values for the association between SNP rs12143842 and IVT is even more significant (*OR* = 0.76(0.65~0.89); *P-obs* < 0.001; *P-adj* = 0.001; P-*emp* = 0.002; Table 2). When the combined group was divided into the ILVT group and the RVOT group, the association remained significant for the RVOT group with *P* values of 0.005 (*OR* = 0.72 (0.55~0.95)).

**Table 2:** Allelic association of rs12143842 with IVT in the Northern and Central cohorts.

Region	Sample size		T allele frequency		*OR* (95% *CI*)	*P*	*OR* (95% *CI*)^#^	*P* ^#^	*P* ^$^
	Case	Control	Case	Control					
Northern	277 (27.56%)	728 (72.44%)	0.28	0.35	0.73 (0.58~0.90)	0.003	0.71 (0.52~0.96)	0.024	0.033
Central	301 (27.26%)	803 (72.74%)	0.29	0.33	0.80 (0.64~0.99)	0.045	0.78 (0.62~0.97)	0.029	0.022
Combined									
Total	578 (27.41%)	1531 (72.59%)	0.28	0.34	0.76 (0.65~0.89)	<0.001	0.76 (0.64~0.90)	0.001	0.002
RVOT	342 (18.26%)	1531 (81.74%)	0.27	0.34	0.72 (0.55~0.95)	0.020	0.60 (0.41~0.86)	0.005	0.004
ILVT	236 (13.36%)	1531 (86.64%)	0.28	0.34	0.75 (0.56~1.00)	0.057	0.70 (0.48~1.04)	0.076	0.066

^#^Adjusted by age, sex.

^$^Empirical *P* values were derived by performing 1,000 Monte–Carlo simulations.

### Genotypic association between SNP rs12143842 and IVT

We found statistically significant genotypic associations between rs12143842 and IVT in both cohorts. The association was more significant with the assumption of a dominant or additive model (*P* = 0.010 and 0.013, respectively) than a recessive model (*P* = 0.029) in the Northern cohort (Table 3). In the Central cohort, the dominant and additive models were significant (*P* = 0.012 and 0.038, respectively). For the combined Gene ID Northern and Gene ID Central cohort population, the *P* values for genotypic association became even more significant for the dominant model (*P* < 0.001). To adjust for potential confounding factors, we performed a multivariate logistic regression analysis. Both allelic and genotypic association between SNP rs12143842 and IVT remained highly significant after adjusting for age and gender (Tables 2 and 3). Empirical *P* values were estimated by performing 1000 Monte-Carlo simulations and were significant (Tables 2 and 3).

**Table 3:** Genotypic association of rs12143842 with IVT.

Region	Case			Control			Model	*OR* (95% *CI*)	*P*	*OR* (95% *CI*)^#^	*P* ^#^	*P* ^$^
	CC	CT	TT	CC	CT	TT						
Northern	143	114	20	310	331	87	Co-dominant					
	(51.62)	(41.16)	(7.22)	(42.58)	(45.47)	(11.95)	CT *vs* CC	0.75 (0.56~1.00)	0.049	0.65 (0.43~0.98)	0.041	0.045
							TT *vs* CC	0.50 (0.29~0.84)	0.009	0.54 (0.27~1.10)	0.090	0.082
							Dominant					
							TT + CT *vs* CC	0.69 (0.53~0.92)	0.010	0.63 (0.42~0.93)	0.021	0.027
							Recessive					
							TT *vs* CT + CC	0.57 (0.35~0.95)	0.032	0.67 (0.34~1.33)	0.251	0.227
							**Additive**	**0.72 (0.58~0.90)**	**0.003**	**0.70 (0.51~0.95)**	**0.023**	**0.022**
Central	137	96	26	301	313	73	Co-dominant					
	(52.90)	(37.07)	(10.04)	(43.81)	(45.56)	(10.63)	CT *vs* CC	0.67 (0.50~0.91)	0.011	0.65 (0.47~0.88)	0.006	0.004
							TT *vs* CC	0.78 (0.48~1.28)	0.328	0.76 (0.46~1.25)	0.275	0.301
							Dominant					
							TT + CT *vs* CC	0.69 (0.52~0.93)	0.013	0.67 (0.50~0.90)	0.007	0.008
							Recessive					
							TT *vs* CT + CC	0.94 (0.59~1.51)	0.792	0.93 (0.58~1.49)	0.753	0.732
							**Additive**	**0.80 (0.64~1.00)**	**0.046**	**0.78 (0.62~0.98)**	**0.030**	**0.025**
Combined	280	210	46	611	644	160	Co-dominant					
	(52.24)	(39.18)	(8.58)	(43.18)	(45.51)	(11.31)	CT *vs* CC	0.71 (0.58~0.88)	0.002	0.68 (0.54~0.86)	0.001	<0.001
							TT *vs* CC	0.63 (0.44~0.90)	0.011	0.65 (0.44~0.96)	0.030	0.023
							Dominant					
							TT + CT *vs* CC	0.69 (0.57~0.85)	<0.001	0.67 (0.54~0.84)	<0.001	0.001
							Recessive					
							TT *vs* CT + CC	0.74 (0.52~1.04)	0.081	0.78 (0.54~1.13)	0.192	0.195
							**Additive**	**0.76 (0.65~0.89)**	**<0.001**	**0.76 (0.64~0.90)**	**0.001**	**0.002**

^#^Adjusted by age, sex.

^$^Empirical *P* values were derived by performing 1,000 Monte–Carlo simulations.

### Left ventricle eQTLs to associate rs12143842 and NOS1AP expression

**Figure 1:**
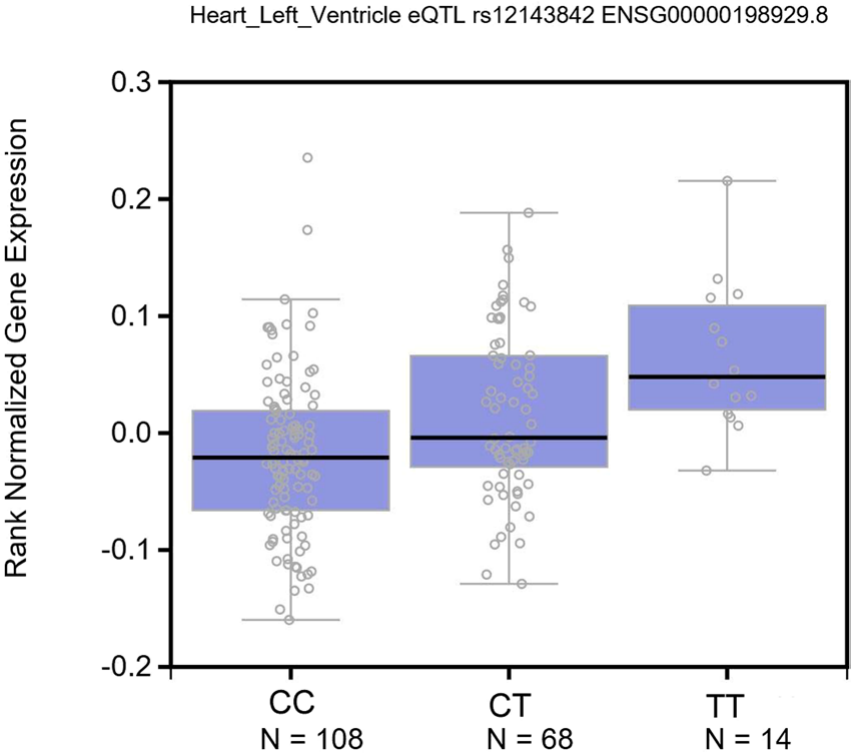
eQTL analysis using the GTEX database (www.gtexportal.org/). NOS1AP expression in the left ventricle was significantly higher with the rs12143842 T allele than the C allele (*P* = 1.4 × 10^−6^, Effect Size = 0.29).

We conducted an expression quantitative trait locus (eQTL) analysis using the GTEX database (www.gtexportal.org/). The T allele of rs12143842 was significantly associated with NOS1AP expression in the left ventricle (*P* = 1.4 × 10^−6^, Effect Size = 0.29) (Fig. 1).

## Discussion

In this study, we show that SNP rs12143842 in the NOS1AP gene on chromosome 1q23.3 is associated with IVT in a non-European ancestry population. We performed a case control association study involving 578 Chinese IVT patients and 1,531 non-IVT controls, all from a large Chinese Gene ID population of Han ethnic descent. For power analysis, we utilized the known population parameters of an OR of 0.70 and T allele frequency of 0.35 in Hap Map. Our study provides statistical power of 90.9% and 89.0% at a Type I error rate of 0.05 in the Northern cohort and Central cohort, respectively, which suggests that our sample size provided sufficient power to identify the association between SNP rs12143842 and IVT. A highly significant allelic association was identified with an OR of 0.76 (*P-adj* = 0.001) (Table 2). Similarly, genotypic association was also significant with an additive model (*P-adj* = 0.001) and a dominant model (*P-adj* < 0.001) (Table 3). These results support the association between the NOS1AP variant and IVT in a Chinese Han population.

To our knowledge, this is the first report to show that the SNP rs12143842 in NOS1AP increases IVT risk. To investigate whether the newly identified IVT SNPs are an extension of the previously reported LD region, we constructed the linkage disequilibrium pattern of a 20 kb 1q23 region including the SNP rs12143842 based on data from 1000 genomes (http://www.ensembl.org, 1000GENOMES: phase_3 CHB and CHS, Supplementary Figure 1). The results showed that rs12143842 derived from any LD block.

The molecular mechanism by which the intronic SNP rs12143842 in NOS1AP increases IVT risk of IVT is unknown. An expression quantitative trait locus (eQTL) analysis indicated that the rs12143842 T allele was significantly associated with NOS1AP expression in the left ventricle (*P* = 1.4 × 10^−6^). Because rs12143842 is located in an intron, one possibility is that it affects the NOS1AP expression level by modulating NOS1AP transcriptional regulation or pre-mRNA splicing, as such regulatory roles have been observed for intronic sequences^11^. NOS1AP encodes the CAPON protein, which is expressed in the heart and exerts its biological effects by interacting with *NOS1* ^12, 13^. NOS1AP was found to be a regulatory factor for L-type calcium channels and the activation of delayed rectifier potassium channels^14^, and overexpression of NOS1AP can shorten action potential duration^14, 15^. Parikh V *et al*. identified that the sustained inhibition of NOS1 increases vulnerability to reperfusion-induced VT/VF^16^. Therefore, we hypothesized that the NOS1AP variant might increase IVT risk by affecting NOS signalling and ion channels, but future investigations are needed to test this hypothesis.

There are several limitations to this study. One limitation is that this is the first time that SNP rs12143842 was found to be associated with IVT in the Chinese Han population; therefore, this finding requires further replication in additional independent Chinese Han populations. Second, the control group was selected from individuals receiving annual physical exams that suffered from hypertension, CAD, stroke, and diabetes mellitus. This might cause selection bias in the control group. Third, the findings in this study might not be applicable to other Chinese ethnicities or to IVT in general due to a selection or referral bias. Moreover, the significant SNP rs12143842 associated with IVT might not be causal, and the functional relationship between the genomic region and IVT is unknown.

In conclusion, we found that the T allele of the SNP rs12143842 in the NOS1AP gene on chromosome 1q23 increases the risk of IVT significantly in the Chinese Gene ID population. The results provided the first piece of evidence that NOS1AP is involved in susceptibility to IVT. These observations suggest plausible mechanisms that might explain our findings in this study.

## Electronic supplementary material

Supplementary Information
