# Supplementary material for: The genetic variation rs12143842 in NOS1AP increases idiopathic ventricular tachycardia risk in Chinese Han populations
Source: Sci Rep. 2017 Aug 21;7:8356. doi: 10.1038/s41598-017-08548-z (PMC5567283; doi:10.1038/s41598-017-08548-z)

Supplementary file information

Article name:

The genetic variation rs12143842 in NOS1AP increases idiopathic ventricular tachycardia risk in Chinese Han populations

Journal name:

Scientific reports

Authors' names:

Ronfeng Zhang Feifei Chen Honjiu Yu Lianjun Gao Xiaomeng Yin Yingxue Dong Yanzong Yang Yunlong Xia

Corresponding address:

Yunlong Xia, MD
First Affiliated Hospital of Dalian Medical University,
Dalian, Liaoning 116011
PR China
E-mail: yunlong_xia@126.com

Yanzong Yang, MD
First Affiliated Hospital of Dalian Medical University,
Dalian, Liaoning 116011
PR China
E-mail: yyzheart@126.com

Supplementary Table 1 Genotypic distribution of SNP rs12143842 genotypes in the GeneID study population

	Characteristic (case/control)	Genotype (case)			Genotype (control)			
		C/C	T/C	T/T	C/C	T/C	T/T	HWE P^a
IVT	Total	280	210	46	611	644	160	0.949
	North	143	114	20	310	331	87	0.883
	South	137	96	26	301	313	73	0.808

HWE, Hardy-Weinberg equilibrium; IVT, idiopathic ventricular tachycardia

[a]HWE P<0.05 denotes the deviation from Hardy-Weinberg Equilibrium

Supplementary Table 2 Compare with allele/genotype frequencies for rs12143842 in all East Asian populations and in the study cohorts

**Frequencies（%）**	East Asian Cohort[&]	Study Cohort	*P*
**Allele（C/T）**	65.9/34.1	67.7/32.3	>0.05
**Genotype(CC/CT/TT)**	42.9/46.0/11.1	42.4/40.5/17.3	>0.05

&: allele/genotype frequencies date for rs12143842 in all East Asian populations was from http://www.ensembl.org/

Supplementary Figure 1 The linkage disequilibrium(LD) pattern of NOS1AP including rs12143842 from CHB and CHS populations

CHB

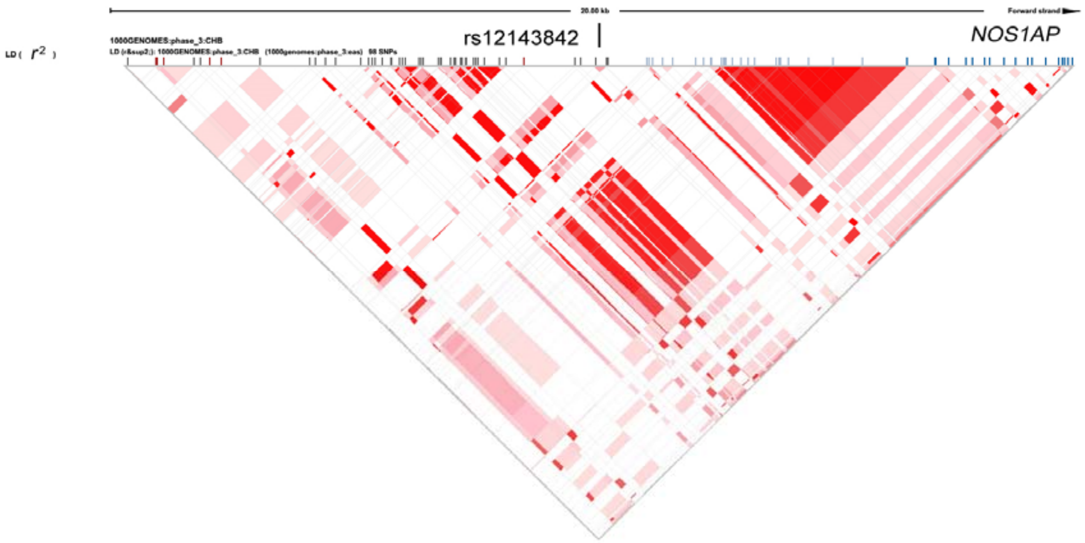

CHS

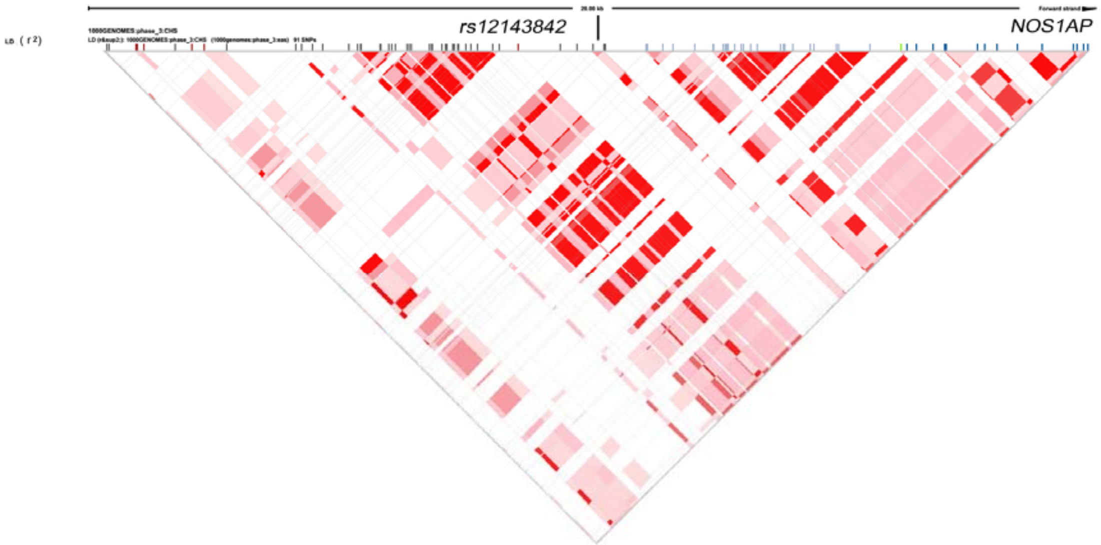

Supplement: Supplementary file 1 — Supplementary Information [file 41598_2017_8548_MOESM1_ESM.pdf]
